# Subcutaneous Emphysema in Patients With COVID-19 Infection: A Report of Three Cases

**DOI:** 10.7759/cureus.10559

**Published:** 2020-09-20

**Authors:** Christian C Toquica Gahona, Kavin Raj, Keshav Bhandari, Shashank Nuguru, Amar Bukhari

**Affiliations:** 1 Internal Medicine, Saint Peter’s University Hospital, New Brunswick, USA; 2 Pulmonology, Saint Peter’s University Hospital, New Brunswick, USA; 3 Pulmonary Critical Care, Saint Peter’s University Hospital, New Brunswick, USA

**Keywords:** subcutaneous emphysema, covid-19, sars-cov-2, acute respiratory distress syndrome [ards], critical care

## Abstract

Subcutaneous emphysema is a rare complication of severe acute respiratory syndrome coronavirus 2 (SARS-CoV-2) pneumonia that should prompt immediate attention to find its cause. Herein, we describe three patients with SARS-CoV-2 pneumonia who were admitted to the ICU and developed subcutaneous emphysema and one with a concomitant pneumothorax.

Three patients with diagnosis of SARS-CoV-2 pneumonia admitted to the ICU developed subcutaneous emphysema during the hospital admission. One of them who had concomitant pneumothorax required thoracostomy tube for treatment and the other two were monitored clinically without additional interventions. Two patients died during the first two to three weeks of their hospital course. One patient survived and was discharged after 63 days in the hospital.

Subcutaneous emphysema is considered a non-life-threatening condition and is usually self-limited requiring supportive treatment in mild cases. For such cases, observation is appropriate. Patients with newly discovered SE life-threatening pathology, such as pneumothorax, esophageal rupture, and necrotizing infections, should be investigated depending on the clinical setting.

This is one of the first paper that shows the development of subcutaneous emphysema in patients with SARS-CoV-2 pneumonia. This may represent a rare complication of the infection as well as may be attributable to other factors such as increased cough and mechanical ventilation. There is a need for studies on the clinical characteristics of a disease with still many unknown features and a wide clinical spectrum that is still being defined.

## Introduction

Coronavirus disease 2019 (COVID-19) is an infectious disease first reported in the Chinese province of Wuhan in December 2019. It is caused by the 2019 novel coronavirus (severe acute respiratory syndrome coronavirus 2 [SARS-CoV-2]) and has been recently declared a global pandemic by the World Health Organization. In the United States, the number of cases has exceeded 3.9 million with more than 142,000 deaths as of July 2020 [1]. The most common clinical presentation of the patients infected with SARS-CoV-2 includes fever, fatigue, dry cough, dyspnea, and diarrhea. After the first week of symptoms, patients may develop a severe clinical state with worsening dyspnea and hypoxemia that rapidly progresses to acute respiratory distress syndrome (ARDS), septic shock, hypercoagulable states, renal failure, and other abnormalities [2].

Subcutaneous emphysema (SE) occurs when gas or air infiltrates in the subcutaneous layer of the skin. The most common causes include recent trauma, surgical procedures, pneumothorax, barotrauma, and infections. On investigations, chest X-ray (CXR) and CT scan may provide identification of intermittent areas of radiolucency or dark pockets in the subcutaneous tissue indicating the presence of gas [3]. 

Despite the great number of publications on clinical features and outcomes of critical ill patients with SARS-CoV-2 infection, complications such as SE and pneumothorax have been rarely described [4,5]. 

We are presenting three patients who developed SE in the setting of SARS-CoV-2 pneumonia and will provide an insight of the clinical features of patients and its implication in management.

## Case presentation

Case 1

A 61-year-old Caucasian woman presented to the emergency room (ER) with eight days of fever, fatigue, dry cough, nausea, and vomiting after a recent trip on a cruise. She had no history of smoking, and her past medical history included gastroesophageal reflux disease (GERD), hypertension (HTN), and type 2 diabetes mellitus (DM). On presentation, her SaO_2_ was 65% on room air and required supplemental oxygen by high flow nasal cannula (HFNC) at 40 L/minute and 100% oxygen (FiO_2_ of 1.0). With this, her SaO_2_ improved to 92%. The CXR showed diffuse patchy bilateral airspace opacities, and nasopharyngeal swab tested for SARS-CoV-2 RNA amplification resulted positive. 

Due to increasing oxygen requirements, the patient was intubated on day 2. The patient received treatment with hydroxychloroquine, azithromycin, ceftriaxone, and methylprednisolone 40 mg IV twice a day for five days.

**Figure 1:**
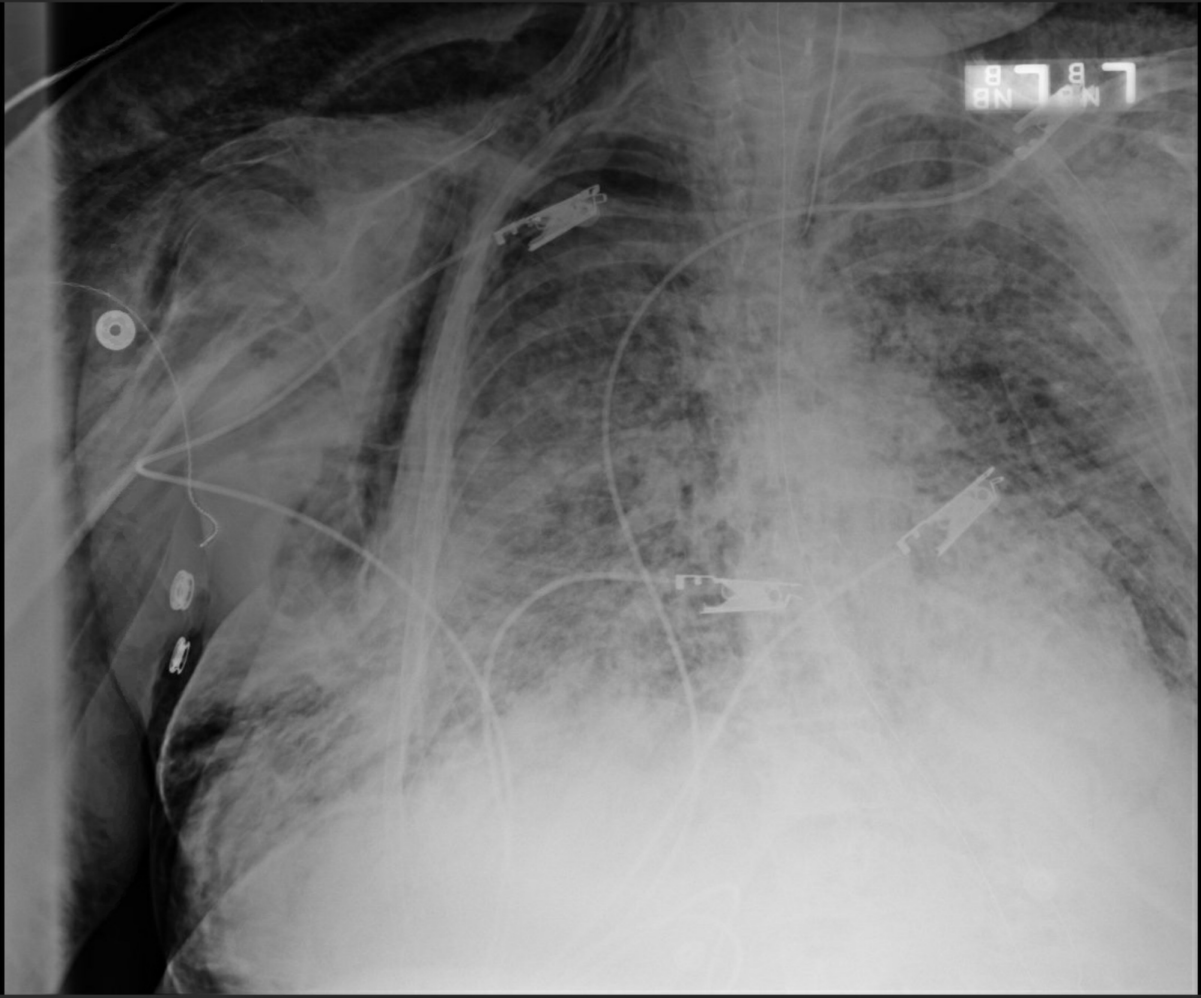
Chest X-ray shows bilateral lung infiltrates and diffuse subcutaneous emphysema in the subcutaneous tissues of the chest.

On day 6 of admission, the patient was noticed to have extensive SE of the chest and neck while she was on ventilator settings: volume control (VC) mode, respiratory rate (RR): 24 bpm, tidal volume (TV): 450 cc, FiO_2_: 50%, positive end-expiratory pressure (PEEP): 13 cmH_2_O. Repeated CXRs showed right-sided apical pneumothorax (Figure 1) that was treated with chest tube thoracostomy with resolution. The patient had ARDS from SARS-CoV-2 pneumonia and developed septic shock and renal failure requiring dialysis. Eventually, the patient passed away on day 18 after admission. 

Case 2

**Figure 2:**
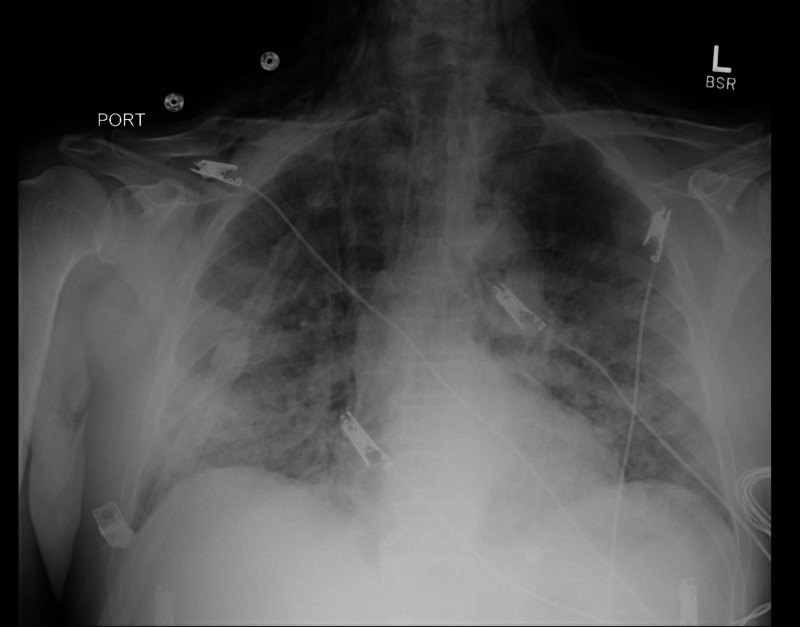
Chest X-ray shows bilateral pulmonary infiltrates and subcutaneous emphysema along the lower neck and upper lung apices.

A 64-year-old male admitted with two weeks of non-productive cough, fever, and chills. He did not have any underlying medical conditions and was a non-smoker. On arrival, SaO_2_ was 76% on room air and he required oxygen through HFNC 40 L/minute and O_2_ at 100% with improvement in SaO_2_ to 95%. CXR showed diffuse bilateral patchy airspace opacities indicative of multifocal pneumonia (Figure 2), and he had a positive test for SARS-CoV-2.

The patient was treated with hydroxychloroquine, azithromycin, and methylprednisolone for five days and piperacillin/tazobactam for seven days.

On a follow-up CXR on day 3 of admission, SE was seen along the lower neck and upper lung apices with no pneumothorax while on HFNC 40 L/minute and 100% O_2_. 

The patient did not require any interventions. The patient had ARDS from SARS-CoV-2 pneumonia and was intubated on day 5 of admission. He eventually developed septic shock and acute renal failure. The patient succumbed on day 14 of hospitalization. 

Case 3

A 30-year-old female presented to the ER after five days of dry cough, sore throat, fever, worsening shortness of breath, and multiple episodes of diarrhea. The patient was diagnosed with type 1 DM since age 13 years and never smoked. On arrival, she was hypoxic with initial improvement on NC 6 L/minute but overnight required HFNC at 40 L/minute and O_2_ at 100%. Her CXR showed bilateral multifocal infiltrates. Her SARS-CoV-2 test was positive. The patient required intubation due to impending respiratory failure one day after she was admitted. 

She was treated with azithromycin and IV methylprednisolone for four days, hydroxychloroquine for five days, ceftriaxone for three days, cefepime for five days, piperacillin/tazobactam for seven days, fluconazole for five days, and micafungin for seven days. Other treatment included one dose of tocilizumab (Actemra®) and one unit of COVID convalescent plasma transfusion. 

**Figure 3:**
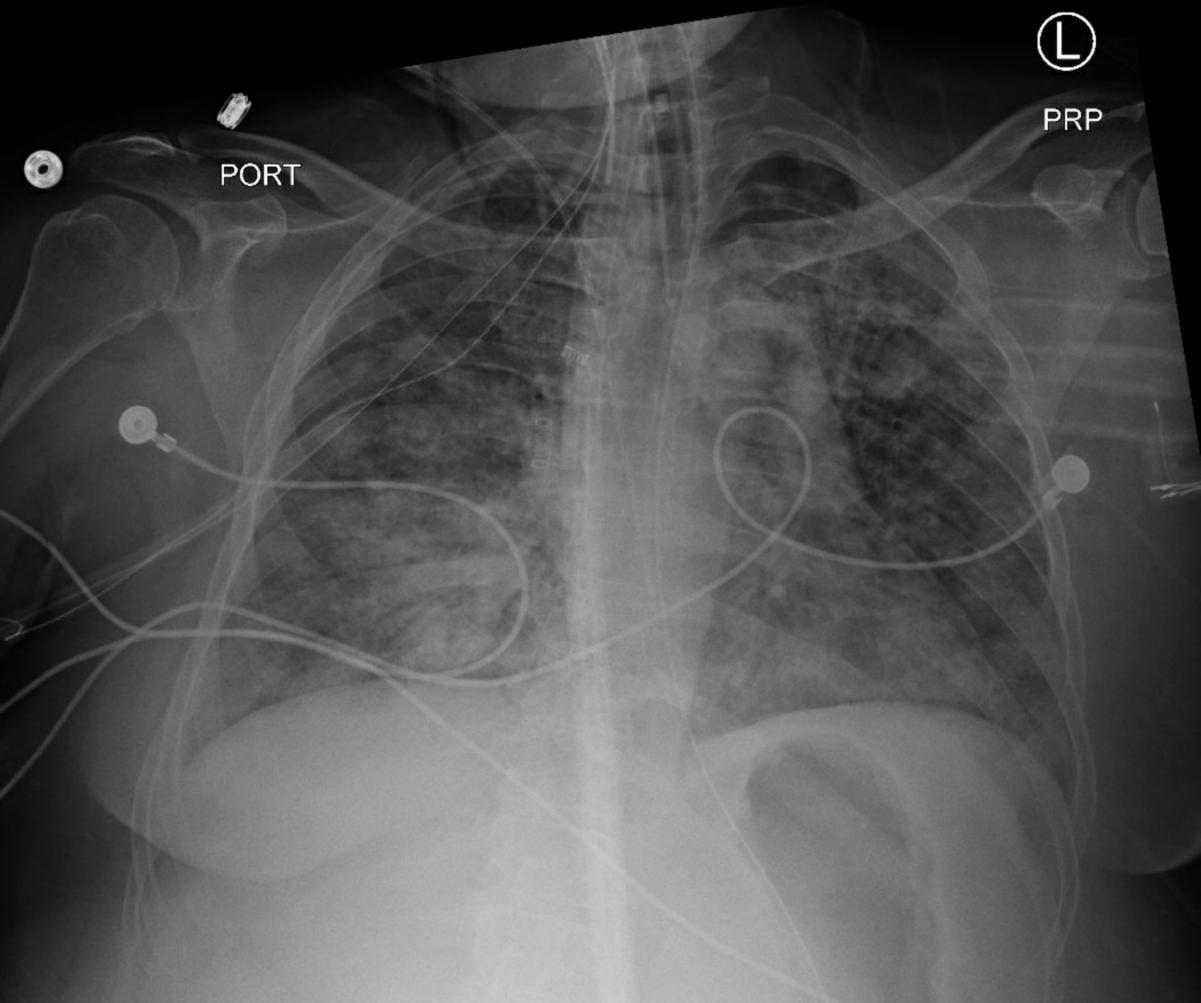
Chest X-ray shows bilateral lung infiltrates and radiolucency in the right side of the neck representing subcutaneous emphysema.

The patient was found to have a new SE in the right side of the neck on day 9 of hospital admission on ventilator settings: VC mode, RR: 25 bpm, TV: 350 cc, FiO_2_: 60%, PEEP: 14 cmH_2_O. A CXR confirmed the findings (Figure 3), it resolved with no need for chest tube placement after seven days. The patient had a prolonged intubation course and underwent tracheostomy on day 28 of hospitalization. The patient was eventually discharged on day 63 of admission. 

## Discussion

Although rare, SE is usually considered a non-life-threatening condition and is usually self-limited requiring supportive treatment. Death from SE is rare and has been reported in the context of pneumothorax. However, clinical significance is important when the amount of air is large or rapidly inflating, as it can place severe pressure on the airway and becoming life-threatening [6]. In patients with newly discovered SE, life-threatening pathology, such as pneumothorax, esophageal rupture, and necrotizing infections, should be investigated depending on the clinical setting. 

Our ventilation strategy for the patients with ARDS included low TV (~6 mL/kg of predicted body weight), the use of high PEEP for alveolar recruitment, and a target plateau pressure ≤30 cmH_2_O for the prevention of barotrauma.

In our patient population, pronounced cough may have promoted alveolar rupture as a result of diffuse alveolar injury in severe SARS-CoV-2 pneumonia. This alveolar damage may have predisposed the patient’s lung to rupturing and generating complications such as pneumothorax and SE, conditions also described in patients with ARDS from other etiologies during mechanical ventilation [7]. The question of whether the SARS-CoV-2 infection predisposed our patients to develop SE is difficult to answer. Two of the patients received conservative measurements and one of them was treated with chest tube placement for an associated pneumothorax.

There was no direct evidence of hemodynamic instability from the SE seen in any of the patients, and the decision of chest tube placement was based on the presence of pneumothorax in the patient who was on mechanical ventilation. We highlight this rare clinical scenario and emphasize that it is vital to investigate the cause of SE in each patient with rapid deterioration to identify diagnoses such as underlying pneumothorax or pneumomediastinum, conditions that may be deleterious for the patients. 

## Conclusions

This is one of the first paper that shows the development of SE in patients with SARS-CoV-2 pneumonia. This may represent a rare complication of the infection as well as may be attributable to other factors such as increased cough and mechanical ventilation. SE is a rare complication that mandates prompt investigation of etiology. Clinicians should be aware that SE may be the first clinical finding that may lead to the diagnosis of life-threatening conditions, such as pneumothorax, esophageal rupture, and necrotizing infections. Both diagnosis and treatment protocols in SARS-CoV-2 patients are in constant and rapid evolution. There is a need for studies on the clinical characteristics of a disease with still many unknown features and a wide clinical spectrum that is still being defined.
